# Obstacles to the prevention of overweight and obesity in the context of child health care in Sweden

**DOI:** 10.1186/1471-2296-14-143

**Published:** 2013-09-30

**Authors:** Gabriella E Isma, Ann-Cathrine Bramhagen, Gerd Ahlstrom, Margareta Östman, Anna-Karin Dykes

**Affiliations:** 1 Department of Care Sciences, Malmö University, Faculty of Health and Society, Malmö, Sweden; 2 Lund University, Faculty of Medicine, The Vårdal Institute (The Swedish Institute for Health Sciences), Lund, Sweden; 3 Skånes University Hospital, Malmö, Sweden; 4 Department of Health Sciences, Lund University, Lund, Sweden; 5 Department of Health Sciences, Health Sciences Centre (HSC), Baravägen 3, Box 157, SE-221 00 Lund, Sweden

**Keywords:** Child, Nurses, Obesity, Overweight, Perceptions, Preventive work, Primary care, Qualitative research

## Abstract

**Background:**

Overweight and obesity in younger children could better be brought in focus through a deeper understanding of how Child Health Care nurses (CHC-nurses) perceive their work with the problems of overweight at the CHC Centers. The aim of this study was to elucidate the CHC-nurses conceptions of their preventive work with childhood overweight and obesity in Child Health Care.

**Method:**

A qualitative study, based on open-ended interviews, involving 18 CHC-nurses strategically selected from 17 CHC Centres in the southern part of Sweden using a phenomenographic approach.

**Results:**

Two categories of description emerged from the data: (i) *Internal* obstacles to the CHC- nurses’ work with overweight in children and (ii) *External* obstacles to the management of overweight in children. The CHC-nurses conceived their work with overweight in Child Health Care to be complicated and constrained by several obstacles depending on the nurses’ personal priorities, knowledge, responsibility and the absence of resources and cooperation, as well as the lack of uniform guidelines for preventing and managing childhood overweight and further a deficient management organisation.

**Conclusion:**

Nurses’ attention to monitoring overweight in children, and their initiative for prevention, is based on their conceptions of the obstacles that hinder them in their efforts. An increased awareness of the CHC-nurses conceptions of the priorities, their sense of responsibility and prevention practices is warranted. If measures in this direction are not taken there is a growing risk that overweight children will pass through the CHC without any formal recognition of their situation. There is an indication that the present level of the CHC-nurses’ preventive work with childhood overweight has room for improvement in several areas. It is suggested that the specialist education of these health care professionals should be supplemented and that organisation of the management of childhood overweight should be also revised at the primary health care level.

## Background

The worldwide prevalence of childhood overweight and obesity has increased during the last two decades and is estimated to affect 60 million children by 2020 [1]. Overweight and obesity in adults is associated with several serious health issues such as cardiovascular diseases and diabetes which subsequently can lead to an earlier death. There’s also a growing movement towards the same negative development among the younger population [2,3]. In Sweden alone, 17% of the 7-to-9-year olds are overweight, including 3% who are obese [4]. Data published 2009-2010 indicates that the rate of overweight and obesity among children in Sweden might have reached a plateau [5,6]. Nevertheless, of today, there exist no national data covering the incidence of overweight and obesity in pre-school children, but there is some regional data available. The regional data from Skåne, the southernmost county in Sweden show that in 2011, 10.1% of 4-year-old children had a high BMI level, divided into 7.8% overweight and 2.3% obesity. During the same period the adjacent county of Blekinge recorded 11.4% overweight and obesity and the county of Uppsala in central Sweden recorded 11.6%. However, among the local municipalities in Skåne there is a wide spread of overweight among 4-year- olds varying from by 4 to 5 times between them [7].

Moreover, current research continues to emphasise the need for effective interventions preferably beginning as early as infancy [1].

Despite the known risks related to childhood obesity there is a deficiency in the implementation of overweight prevention within primary care which has resulted in a situation where younger children with overweight and those with moderate overweight often pass through the system unrecognised and without action being taken [8].

CHC in Sweden is free of charge and it is actively offered to every pre-school child [9]. Registered Sick Children’s nurses (RSCNs) and District nurses (DNs) i.e. Child Health Care nurses (CHC-nurses) are placed at CHC centres to provide preventive care and counseling for families in general. The role of the CHC-nurses is to detect and prevent illness and risks to children from birth to the age of six years [9]. Therefore as most children pass through the CHC it is a very suitable catchment point for supporting parents in an active parenting, thereby promoting children’s health, safety and development.

Hence, the focus on the CHC-nurses’ prevention practices is of great importance since they have the possibility to recognise a child’s eventual overweight problems and have the potential to prevent further weight gain that is caused by an unhealthy lifestyle that leads to obesity at an earlier stage in life. There are some studies conducted in the UK and the USA relating to various other Health Care Professionals’ (HCPs *excluding* RSCNs and DNs in CHC), attitudes [10], views [11-13] and beliefs [14] of managing [10,13], treating [12,15] and preventing [11,14,15] childhood obesity in primary care. However, to the best of our knowledge research is scarce about how CHC-nurses perceive their work with childhood overweight at the CHCs.

Raising awareness at the correct level in the Primary Care Services, of the CHC nurses’ perceptions of the present methods available for the prevention of childhood overweight could shed light on those areas that need to be addressed. This in turn might enable CHC-nurses to identify and put forward possible ways of improving their work, at the CHC, in the area of child overweight. A previous study has shown that paying attention to the CHC-nurses’ conceptions of overweight in children is important since the nurses’ conceptions might affect the parent-nurse relationships and thereby the nurses’ and the parents’ efforts to influence children’s weight [16]. The management of younger children with overweight could be improved by the Primary Care Management having a better understanding of how CHC-nurses perceive their work with childhood overweight.

The aim was to elucidate and describe how CHC-nurses conceive their work involving the prevention of childhood overweight and obesity in CHC.

## Methods

A phenomenographic approach was chosen for the interview study outline. The focus in this approach is on the variety of human ways of understanding the world around themselves. The use of interviews is a common way to collect data [17]. Transferred to this study, it means that the CHC-nurses’ varied ways of understanding preventive work with childhood overweight at the CHC could be captured. From the nurse’s individual interview statements their different conceptions of their work with overweight children could be identified and formed into categories of description in an analysis. These categories of description represent a collective level of the various conceptions [17].

### Participants

The professional background of a CHC-nurse is either a RSCN or a DN. RSCNs are registered nurses with specialisation in primary health- and medical care for children, adolescents and their families, in contrast to DNs who are registered nurses with some type of specialisation in primary health- and medical care for children but with focus on adults and the elderly.

Overall, 91 CHC-nurses were eligible for this study at 26 CHCs, located in four municipalities in the southern region of Sweden. A strategic selection of both CHCs and CHC-nurses was made in order to ensure the possibility of collecting a broad range of conceptions. A total of 17 CHCs were stratified according to their catchment area in order to get a variation of citizens regarding socio-economic and ethnic backgrounds. The CHC-nurses were stratified according to age, gender, nationality, years in their profession and years in CHC. The professional backgrounds of the CHC-nurses are RSCNs and DNs with experience of working with children (0 - 6 years) for at least one year including children who are overweight.

The decision to stop recruiting further nurses for this study was taken at the point when no new information was added and no new conceptions emerged. Finally, 18 CHC-nurses from 17 CHCs constituted the participants in this study. Two of the participants came from the same CHC centre. Originally, 19 RSCNs and DNs were invited to participate, however, one nurse declined due to a heavy workload. The mean age of the nurses was 51 years (range 32-63 year) with a mean of 11 years (range 1-29 year) in CHC (Table 1).

**Table 1:** Demographic characteristic of CHC -nurses (n = 18)

**Characteristics**	**CHC-nurses (n=) [%]**
***Gender***	
Male	1 [6]
Female	17 [94]
***Age***	
30 - 50	9 [50]
51 - 65	9 [50]
***Country of birth***	
Sweden	15 [83]
Other	3 [17]
***Professional background***	
RSCN	10 [55]
DN	5 [28]
DN + RSCN	3 [17]
***Years in CHC***	
1 - 10	11 [61]
11 - 20	6 [33]
21 - 30	1 [6]

### Procedures and data collection

Written agreement to carry out the study was obtained from the local director of the Primary Health Care who was personally informed about the study*.* The responsible manager at each CHC centre was then formally informed about the study by the director of the Primary Health Care. Further, using a staff list, the first author initially approached a number of presumptive participants by e-mail and then two weeks later by telephone.

The interviews were carried out by the first author from December 2010 to March 2011. All of the interviews took place at the offices of the participants during their working hours. All of the participants were given written and oral information and were requested to give their written informed consent after which the interviews were performed, digitally recorded and then transcribed verbatim.

#### *Interviews*

This study was the second part of an interview study with CHC-nurses concerning childhood overweight in CHC. The first part covered the CHC-nurses conceptions of childhood overweight [16]. An interview guide with open-ended questions was developed, relating to the aim of this study. The introductory question in the interview was: *What is your perception, as a nurse, of the preventive work against childhood overweight and obesity in CHC?*

The interviews were held in conversation form and depending on the character of the answers, the responses were explored using further questions. The interview guide included supplementary questions such as; *what do you mean? Can you clarify? Can you develop your thoughts? In what way? Can you exemplify? Is there anything you want to add?* The median length of the total of the interviews was 46 minutes, ranging between 26 to 90 minutes.

### Data analysis

The analysis was carried out in five-stages using the phenomenographic approach [18-20].

Stage 1 To ensure that the interviews were transcribed correctly, the transcripts (n = 18) were all listened to by the interviewer and read through several times by three of the authors (GEI, AKD and ACB) in order to gain an overall sense of the content*.*

Stage 2 The statements relevant to the study was identified according to each of the participating nurse’s different ways of perceiving the preventive work on childhood overweight and obesity in the context of CHC. The statements were continuously compared and sorted into different preliminary conceptions.

Stage 3 The identified conceptions were then compared on the basis of their differences and similarities in relation to each other. The conceptions were grouped into preliminary categories of description according to their common features.

Stage 4 The final categories of description were named, in order to emphasise their essence, based on the variety of the conceptions included.

Stage 5 At this stage of the analysis the focus shifted to the relations between the categories of description. The categories of description was finally scrutinised to ensure their coherence with the different conceptions.

### Ethical considerations

Approval for the study was granted by the Regional Ethical Review Board in Lund (Dnr. 2010/694). All of the interviews were voluntary, and were conducted according to the Declaration of Helsinki [21].

## Results

The main findings elucidating CHC-nurses conceptions of the preventive work with childhood overweight in CHC are presented in Table 2. Seven conceptions formed two categories of description; (i) internal obstacles to nurses’ work with overweight in children and (ii) external obstacles to the management of overweight in children. The conceptions in each category of description are described in the following text and are illustrated by selected quotes. Each quote represents one specific interviewee labeled by a numerical code in brackets.

**Table 2:** CHC-nurse’s conceptions of the preventive work with childhood overweight and obesity in the context of child health care

**Categories of description**	**Conceptions**
**Internal obstacles to nurses’ work with overweight in children**	Other priorities than childhood over- weight
	Insufficient knowledge regarding the identification and handling of childhood overweight
	Differences in nurses sense of responsibility and prevention practices
**External obstacles to the management of overweight in children**	Parent’s cooperation-a vital concern
	Absence of resources and cooperation with other agencies
	Deficient management organisation of childhood overweight
	Lack of uniform guidelines

### Internal obstacles to nurses’ work with overweight in children

In this category, the CHC-nurses highlight their own perceived ability to adequately manage cases of childhood overweight. Their conceptions were related to their priorities and their approach, their self-perceived knowledge about the management of childhood overweight and the recognition and assessment thereof.

#### *Other priorities than childhood overweight*

CHC-nurses conceived that despite an obvious proportion of children with overweight noted within the catchment area of their CHC centre, there are not many overweight pre-school children passing though the CHC. According to the interviewees, there are statistics showing a local increase in the prevalence of overweight in CHC, but this fact does not correspond with the nurses’ clinical experience. According to the nurses interviewed, the intensity of prevention practices depends on the prevalence of overweight children passing through the CHC centre. The interviewees conceived that overweight is a phenomenon that one often talks about, but however it is rarely seen at the CHC centres.

The CHC-nurses stated that due to their limited time and resources, there are other issues that they conceive to be more important and requiring higher priority than a child’s overweight. They considered that the prevalence of overweight and especially obesity at their own CHC centre is low and thought that possibly the level of concern was considerably higher in other CHC units. The nurses conceived that CHC centres placed in districts with a large number of immigrants and refugees have a greater proportion of children with overweight and obesity. Their conception was that socio-economically better placed CHC centres do not have any obese children at all and very few overweight children and parents. According to the interviewed, the small proportion of overweight children passing through CHC was not conceived as being a concern in need of a greater effort from the nurses or the CHC. The study participants conceived that the preventive work on childhood overweight in CHC is not considered important. Hence, the CHC-nurses stated that they see each child far too seldom to be able to make a difference that would matter. One of the problems often mentioned by the interviewees as an important reason for concern is underweight, which occurs more frequently and is more closely associated with illness and disease than is overweight.

*“…if we had a lot of overweight children and maybe some with diabetes, then maybe we would have made different priorities and put a larger effort into it. But, because there only occurs one or another overweigh child from time to time, I don’t feel it is worth putting so much energy into it”.* (1:11)

#### *Insufficient knowledge regarding the identification and handling of childhood overweight*

The conception of the CHC participants was that they did not have the skills and the tools to adequately manage overweight and obesity in children. CHC-nurses considered it to be important that they have the specific skills that are required for their encounters with the parents and children. Most of the participating nurses conceived that their education, as well as their in-service training lacked sufficient focus on managing childhood overweight in CHC. They also requested more time and expertise in prevention practices and training in how to tackle different situations. They conceived that their own capacity to influence any negative development of a child’s weight is limited.

*“I can offer to take an extra weight measurement and such, but I have neither the time nor the knowledge to do so very much, really”.* (1:16)

Major deviations in weight in a child’s growth pattern over a short time, in combination with parental obesity, constituted a basis for concern. However, the CHC-nurses conceived that there were several difficulties regarding identifying children with overweight. They considered that it was obvious when a child was obese, especially if the child was older, and the child’s overweight appearance was clearly recognised as deviant. It was considerably more difficult to discern overweight from an acceptable weight, particularly if the child was younger. The interviewees expressed uncertainty about the assessment of BMI and most of them did not know the definitions of overweight or obesity in children.

*“No, I haven’t calculated BMI, partly because I don’t master it well – the BMI in children – and partly because I don’t know the threshold values. Certainly I could have looked it up but then I don’t feel comfortable about informing the parents”.* (1:14)

Consequently, the CHC-nurses stated that the BMI-measurements were rarely considered and that it was more common that the CHC-nurses simply used their visual appraisal to determine if a child was overweight or not.

“… *I do not agree with what I see in front of me. The curves in the chart can show that the children are proportionate but the BMI shows that they are over the threshold values /…/ To be honest I have not bothered to calculate BMI lately. I was very careful at one time, writing down the value in each chart, but lately I have not done this”.* (1:13)

#### *Differences in nurses sense of responsibility and prevention practices*

The participants considered that the CHCs duty is restricted first and foremost to getting the parents to take notice of their child’s overweight problem and later giving the parents the correct and adequate information regarding healthy diets for their child. The nurses’ conception was that the only prevention strategy a CHC-nurse can offer is general advice and that no resolute measures are ever taken by the CHC.

The interviewees conceived that the prevention of childhood overweight is not solely the responsibility of the CHC since there are other actors involved. Those who have the uppermost responsibility for solving a child’s overweight problem are the parents, and to a certain extent, society also shares the responsibility*.* Since children spend most of their time at pre-school eating and playing, the participants conceived that, the pre-schools have a greater responsibility for childhood overweight than the CHC. Some of the nurses stated that on their own initiative, they offered weight assessments of the children as a follow-up to recommendations regarding a healthier diet and also a diet diary, to be kept by the parents, to record their child’s progress. The participants considered that families with overweight children were in need of professional guidance such as that from a; dietician, paediatric clinic and a Child Obesity Unit (COU), and that it was a part of the CHC-nurses responsibility to give the parents support and refer the families to the right unit. According to the participant CHC-nurses, the few children whose overweight is recognised at the CHC are rapidly taken care of. They are preferably referred to either the COU, to the dietician or to the School Health Care (SHC) once they have started school. The referral is often preceded by counseling.

*“We have referred them to the paediatric clinic because it has been said that we shouldn’t take care of overweight children. We are to pass them on”.* (1:12)

### External obstacles to the management of overweight in children

This category summarises CHC-nurses conceptions regarding insufficient support and cooperation, the deficient management organisation of childhood overweight and the lack of uniform guidelines.

#### *Parent’s cooperation a vital concern*

The participants considered that their role at the CHC is very important and they also conceived that a condemnatory approach to informing parents about their child’s weight status has an impact on how the parents receive the nurse’s recommendations. Therefore the participants considered that a good cooperation between the CHC-nurses and the parents regarding the management of childhood overweight is essential. However, no matter how much the nurse informs the parents and how much the nurse engages herself, it still depends much on what the parents choose to do with the information they receive and what they later choose to do about their child’s weight.

*“I can talk and inform, but the step towards doing something isn’t mine”.* (1:7)

The participating CHC-nurses conceived health promotion as a tightrope between motivating parents towards making a positive change and to creating anxiety and insecurity among them. However, the biggest concern among the nurses who were asked for their perceptions concerning their preventive work with childhood overweight in CHC are those parents who despite their initially positive attitude ultimately choose to ignore the nurses’ recommendations and referrals.

*“The Child Health Care has been criticised a lot lately/…/We have to ask the parents what they think about the child’s weight issue and listen to their solutions through mirroring their own thoughts”.* (1:15)

#### *Absence of resources and cooperation with other agencies*

The participants conceived that there are several obstacles that complicate the nurse’s preventive work with overweight children in CHC. The nurses considered that a major flaw is that there is too little cooperation between the CHC and the COU which severely limits the CHC-nurses work. Further, dieticians where described as an important resource but also a resource to which there was now significantly limited access.

*“Sometime we have talked to the dietitian, who replied that they didn’t have the resources to take care of overweight children. They had to focus on the allergic children instead. And the child obesity unit doesn’t receive children of this age”.* (1:2)

The participating CHC-nurses conceived that referrals issued by the CHC are sometimes rejected by the COU, due to workload, the child’s age or the lack of severity of the weight issue. According to the interviewees, this leaves the CHC-nurses isolated and powerless to deal with overweight children and their families. They also noted that they lacked cooperation from the maternity health care, the COU and the pre-schools regarding this issue.

#### *Deficient management organisation of childhood overweight*

The participants noted that they considered the management organisation for overweight children was ambiguous and insufficient*.* The participating CHC-nurses conceived that due to the Primary Health Care unit managers disinterest in CHC they made no effort to properly organise the management of the recourses pertaining to childhood overweight and obesity.

*“…the BMI should have been introduced, but nothing happened and we continued as before. These children cannot be placed anywhere, anyway”.* (1:2)

Moreover, the CHC-nurses conceived that the dominating problems and issues imposed on them by their superiors demanded too much of their attention and reduced the time they had to spend on overweight children and their other duties. The nurses considered, that at the moment, their highest priority regarding the care of children were, the problems caused by parental smoking, mothers with postpartum depression, parental child neglect and child autism.

#### *Lack of uniform guidelines*

The participants conceived that the lack of uniform guidelines for the care of overweight and obese children was a deficiency. Generally, they considered that the CHCs require an approved list of explicit principles related to the management of overweight children. They also conceived that the few guidelines they have are brief, general and unclear and need to be updated. Some of the participant nurses stated that they were not aware of any existing official guidelines regarding the management of overweight children in CHC. The participants all expressed that they conceived that each of their CHC-colleagues tackled the problem in a different way due to the lack of uniform guidelines and instructions.

According to the participants, overweight and obesity prevention practices depend much on the individual CHC-nurse’s focus preferences which can vary from moderate attention, for example, making continuous observations of a child’s diet, physical activity and their eventual weight development, to taking little or no attention. Eventual nurses’ observations could be followed with further questions, information or remarks to the child’s parents about any problem they had observed. Following their discussions with the parents, nurses could, according to their own initiative, follow-up a child’s progress after a couple of months to one year later, by checking the child’s weight and body measurement or making a diet re-evaluation. However, whatever they took the initiative to do in each case was not the result of any specific official guidelines. According to the interviewees, in these cases they carried on in spite of no existing guidelines.

*“Actually, it isn’t included in the base programme to take them back. Because then there would be opportunities to bring them here, actually – I did so with one child when the family refused to go to the child obesity unit. They came to me once a month and we just took the child’s weight each time. Just this little made a difference to this girl’s weight. It reduced a little – when someone watched it”.* (1:4)

## Discussion

### Summary of the main findings

The results of this study present how CHC-nurses’ conceive the preventive work with childhood overweight and obesity with a wide variation of obstacles affecting their work. To summarise, CHC-nurses conceptions were related to perceived obstacles to their work with childhood overweight which are divided into two main aspects; (i) conceptions related to *internal* factors described as the way nurses make priorities, take responsibility and their lack of knowledge. The second aspect (ii), involves conceptions related to *external* factors described as the absence of resources and cooperation, a deficient management organisation related to the handling of childhood overweight in CHC and a lack of uniform guidelines. The descriptions that emerged highlighted important issues which can negatively affect the preventive work with childhood overweight in CHC.

This area has been previously explored amongst various health care professionals but, to the best of our knowledge, has not been conducted specifically related to the CHC-nurses conceptions of the activities surrounding the prevention of childhood overweight and obesity in CHC. Our finding is partly supported by previous research dealing with different health care professionals’ beliefs and knowledge related to preventing and treating childhood obesity [10-15], discussed further below.

#### *Priorities, knowledge and responsibility*

This study shows that CHC-nurses conceived that it is their duty to raise the issue of a child’s overweight with its parents; however, it is mainly the parent’s responsibility to deal with the problem. Similar findings are presented by Walker et al., [12], who describe general practitioners (GPs) and family practice nurses unwillingness to accept the responsibility for the problems of obesity in children due to the difficulties involved in this issue.

Additionally, Redsell et al., [14] reported a low level of concern among health care professionals dealing with young children who may be at risk of developing obesity and that GPs and nurses were not consistently recognising overweight and obesity in younger children. Further, they reported there was a lack of terminology, training and guidance, which made the prevention of overweight among children difficult. This is in line with our findings showing that CHC-nurses perceived their knowledge and skills on the subject to be insufficient. The CHC-nurses in the current study expressed their limited knowledge of handling childhood overweight and their ability to make correct assessments. Moreover, most of the CHC-nurses participating in this study had difficulty in differentiating between overweight and obesity. They were unable to define overweight or obesity in accordance with any specific reference and rarely used the BMI scale for respective age or monitoring those children at risk for obesity. Instead, they used growth charts, weight status and their own visual estimate to define a child’s possible overweight. Turner et al., [13] describe the practitioners own skills and motivation as being barriers as do Story et al., [10] who show that self-reported low proficiency in counseling-related skills are barriers to the management of childhood obesity which is consistent with our findings.

Our results are further supported by Larsen et al., [11] who show that despite awareness of childhood obesity prevention guidelines, most nurses do not regularly use BMI for respective age or monitor those children at risk of obesity. This is likely to affect their ability to identify this category of children and their own perception of the magnitude of the problem. According to O’Brian et al., [8] this might lead to the risk of generally lower recognition of the problem of childhood overweight resulting in under treatment.

There is a contradiction in this study, where nurses state that they see no prevalent problem of overweight in their districts while at the same time pointing to number of obstacles hindering them being able to deal with the problem. After comparing the regional data on 4-year-olds with overweight [7] with the nurses’ perceptions of the prevalence of the problem the authors conclude that there is a lower recognition of childhood overweight at the CHCs. Another explanation for this apparent contradiction could be the nurses´ ability to perceive the magnitude of the problem at their district [16] or that they simply ignore the situation due to workload.

Klein et. al., [22] indicate that training, time and resource limitations affect the use of the BMI-percentile by pediatricians and suggest that the awareness of national guidelines might improve the prevention of obesity, as they would recommend the use of the BMI-percentile. This would also be a useful tool for the CHC-nurses in their work with overweight children.

#### *Lack of resources and cooperation*

Our study shows that nurses working with overweight in CHC conceive that they are left alone by the Health Care system, to deal with this concern, which is a perplexing situation. This is in line with a study by Brown et al., [23] indicating that primary care nurses require proper support from the Health Care system in the practice of dealing with obesity in adults. Our findings also show that the cooperation between the CHC and the Child Obesity Unit is limited and it is sought-after by the nurses. Furthermore, the participants in this study noted that there was a lack of cooperation between the CHCs and the maternity care and pre-schools in this field which is supported by Chamberlin et al., [15] implying the need for developing collaboration with other actors who can have an impact on the treatment of childhood obesity.

Another important concern brought forward in our study was that regarding nurses having to relying on parent’s cooperation to help their children with the problem of overweight. The CHC-nurse participants´ conception was that, on the first hand, it is the parents and the pre-school personnel who have the prime responsibility to monitor children at risk for overweight The CHC-nurses considered it very important that the parents take on the responsibility and follow through the recommendations they are given. This finding is in line with a study by Walker et al., [12] who show that although the GPs and the family practice nurses recognise the importance of the family’s role in solving the issue, they would rather pass on the responsibility to the parents although in fact the parents may be unwilling or unable to accept this responsibility.

The results in this study are to some extent supported by Turner et al., [13] who show that the lack of expertise in how to treat overweight, resources and limited time were barriers, as well as the level of health care contact with children. Limited time for appointments and the rare prevalence of primary school children visiting a surgery were also restrictions to their dealing with childhood overweight according to the GPs and family practice nurses involved.

Further, current study shows that families ignore the nurses’ referrals. Turner et al. also commented that according to the GPs and nurses in their study, most of the patients that were offered follow-up appointments did not return [13]. Some GPs and family practice nurses describe primary care as an unsuitable treatment setting for childhood obesity. Even those who didn’t agree with this had doubts about the possibility to reduce a child’s obesity using some kind of treatment [13].

The main findings of this study show that despite the negative nature of the conceptions of the participants they have a positive attitude towards the prevention of obesity. The participant CHC-nurses asked for more education, increased cooperation with other actors and clearer guidelines, which indicate that there might exist awareness among them of their own limitations. The participants point to a lack of guidelines, but nurses are individuals with professional independence, however, they need to learn how to utilise international research, which they all have access to through the internet at their workplace. According to the CHC nurses, what is missing is time. The recommended base programme for the CHC centres in Sweden is considered by the CHC-nurses as being static, but they have the possibility and the freedom of action to customise it to the individual.

### Methodological considerations

The present study has both strengths and limitations. This interview study used a phenomenographic approach conducted in the specific field of the prevention of childhood overweight and obesity in primary care. Findings generated from phenomenographic research have educational relevance [24] as they can assist the disciplines of nursing by adding knowledge about variations in how the prevention of overweight is experienced in CHC [19]. Perceptions of a phenomenon can affect the behavior of both the health care personnel [16,20] and parents [16]*.* Content-related descriptions of a group of professionals’ perceptions of the prevention of overweight in CHC are a resource for enhancing awareness [19].

From a gender perspective there is a limitation in that the number of male CHC-nurses in the southern part of Sweden is low. However, all those eligible took part in this study, which can be seen as a strength. The professional and gender aspects might have an influence on the findings of this study, but to the best of our knowledge there are, to date, no studies conducted that compare these two aspects in the same study.

The 18 CHC-nurses in this study were from 17 different CHCs, covering those areas where the problem of overweight is reported. The CHCs included were situated in both well-to-do and poorer areas and the CHCs differed in their composition of RSCNs´ and DNs´ as well as in the number of employees per unit. They all had the same local director but each had their own responsible clinical manager*.* This should not have influenced the findings, as they all have the same general guidelines for CHC. However, our findings show individual differences among the nurses which can be interpreted as a result of unclear guidelines and lack of education focusing on preventive work among overweight children. Larsen et al., [11] showed in a study concerning childhood obesity, that there exist differences regarding specialty, practice setting and awareness of the prevention guidelines. These differences were found between advanced family nurse practitioners and advanced paediatric nurse practitioners working in primary family care or primary paediatric care settings. However, the findings of the present study might be transferable to similar contexts in CHC as the sample of the profession represents all nurses employed at CHCs exclusively, and the sample of CHCs represents both urban and rural areas, including those with a large number of immigrants.

The semi-structured interview is the preferred method of data collection in phenomenographic research [25]. In the interview situation it is of value that the interviewer obtains a clear understanding of how the informants understand the phenomena in focus [18]. In this study the participants spoke freely and had a positive attitude toward the interview. The duration of the interviews may have been affected due to the fact that the interviews were held at the CHC-nurses work place and there were sometimes time restraints due to the nurses’ involvement in their work. Following the interviews all but one of the nurses noted their satisfaction with their contribution and had nothing more to add. One participant wished to continue the interview as there had been interruptions and the nurse felt the interview to be unfinished so the interview was completed the next day.

The relationship between the empirical data and the findings of a phenomenographic study is the core question of credibility. By providing the reader with excerpts from the interviews, the relevance of the categories is supported [18]. This was done by the presentation of individual quotes in this study. Further, to strengthen the validation and trustworthiness of the study, the findings were continuously discussed back and forth between the authors during the analysis until a high degree of inter-subjective agreement was reached [18].

## Conclusion

The nurse’s conceptions of their role and their work, along with their knowledge of overweight and existing guidelines at the CHC affects their dedicated time and their priorities. The participating CHC nurses conceive themselves to be professionally unprepared to manage childhood overweight, which can result in their failure to recognise cases of overweight among the younger children in their care.

Absence of resources and the deficient management organisation of the primary health care impede the prevention of childhood overweight at the CHC and the participating CHC-nurses request more cooperation from all those instances who are involved in these children and their families.

This area of research is still unexplored and to the best of our knowledge this is the first study conducted involving CHC-nurses conceptions of the prevention of childhood overweight in CHC. This study adds the conceptions of the CHC-nurses to the accumulated knowledge of the perceptions of Health Care Professionals regarding the prevention of childhood overweight.

### Implications for research, practice, education and organisation

Nurses should be aware of the impact of overweight and obesity even among the younger population. There exist knowledge gaps in the education of specialist nurses in child health care and there is a need to supplement their education. There should be greater focus on childhood overweight in the education of RSCNs and DNs, including further education in CHC regarding the CHC-nurses’ role and responsibility including their contact with the parents. The CHC-nurses need to enhance their ability to observe and take action earlier, that it is to say, before the children reach school-age.

Time, outside the base programme for CHC nurse’s standard duties should be allocated at an early stage to those families with children at risk for obesity. The management of childhood overweight should be developed in the primary health care in order to update and coordinate the work of the health care professionals involved. Overweight requires the attention of all the involved authorities and it is suggested that there is a need for enhanced cooperation throughout the whole health care chain from the Maternity Health Care to the Child Health Care, the School Health Care and the Child Obesity Unit, as well as the Paediatric Clinic in order to meet the goal of a healthier population. The problems involving childhood overweight should therefore be recognised as a priority in CHC and the primary health care.

This study indicate that there is a need for further research that unravel the CHC-nurses somewhat ambiguous perception of overweight and obesity in younger children.

## Competing interests

The authors declare that there are no conflicting interests.

## Authors’ contributions

The design of the study and the interview guide was developed by the first and the last author (GEI and AKD). All of the interviews, as well as the initial analysis of the interview transcripts, were conducted by GEI. The draft and the reporting of the manuscript were also the work of the first author (GEI). AKD and ACB assisted GEI with the analysis of the interview transcripts. The analysis was discussed amongst all of the authors and AKD, ACB, GA and MÖ regularly made comments on the reporting and the draft of the manuscript. GEI obtained the funding. Finally, all of the authors read and approved the submitted manuscript.

## Pre-publication history

The pre-publication history for this paper can be accessed here:

http://www.biomedcentral.com/1471-2296/14/143/prepub
